# Temporary Peripheral Nerve Stimulation (PNS) of the Cervical Medial Branch Nerve (CMBN) for Chronic Axial Neck Pain—A Literature Review and Case Series

**DOI:** 10.3390/jcm14165910

**Published:** 2025-08-21

**Authors:** Vinicius Tieppo Francio, Kelsey Gustafson, Logan Leavitt, Ryan Zwick, Christopher M. Lam, Andrew Sack, Dawood Sayed, Usman Latif

**Affiliations:** 1 Division of Pain Medicine, Department of Anesthesiology, Washington University School of Medicine in St. Louis, 660 S. Euclid Avenue, CB 8054, St. Louis, MO 63110, USA; 2 Department of Anesthesiology and Pain Medicine, The University of Kansas Medical Center, 3901 Rainbow Boulevard, Kansas City, KS 66160, USA; 3 Department of Physical Medicine and Rehabilitation, The University of Kansas Medical Center, 3901 Rainbow Boulevard, Kansas City, KS 66160, USA; 4 Midwest Anesthesia Associates, 9100 West 74th Street, Overland Park, KS 66204, USA

**Keywords:** peripheral nerve stimulation, chronic neck pain, cervical medial branch nerve, neuromodulation

## Abstract

**Background:** Peripheral nerve stimulation (PNS) has been employed as a therapeutic modality for managing chronic pain across diverse etiologies and neural targets. Nevertheless, its application in treating chronic axial neck pain remains markedly underexplored. Accordingly, this study aimed to both review the existing literature and present a retrospective single-center case series of patients who underwent temporary PNS targeting the cervical medial branch nerves (CMBNs) for chronic axial neck pain. **Methods:** This investigation comprises a narrative literature review alongside a single-center, retrospective case series evaluating percutaneous, temporary PNS for the management of cervical spondylosis facet arthropathy in the absence of myelopathy or radiculopathy. The primary outcomes were pain reduction, as measured by the numeric rating scale, and improvements in functional disability, with assessments conducted at baseline and at 60 days post-intervention. **Results:** PNS represents a neuromodulatory, nondestructive intervention that targets the CMBN to alleviate chronic axial neck pain, in contrast to the destructive mechanisms inherent in cervical radiofrequency ablation (CRFA). Although PNS has been applied to other neural targets, its use in the cervical region is sparsely documented, with limited case studies available. Notably, this case series is the first to report pain and disability outcomes specifically associated with CMBN PNS. At the 60-day follow-up, 66% of subjects achieved the minimal clinically important difference (MCID) for pain reduction, while 77% met the MCID for disability reduction. Moreover, our analysis uniquely examined the impact of previous CRFA and a history of cervical spine surgery on treatment outcomes, revealing that patients with such interventions experienced more modest improvements compared to their surgery- and CRFA-naive counterparts. **Conclusions:** The current literature reveals a significant gap regarding the use of CMBN PNS, underscoring an unmet need in the treatment algorithm for chronic axial neck pain beyond conservative modalities. Our findings suggest that CMBN PNS may offer a promising adjunctive therapy for carefully selected patients with refractory chronic axial neck pain who have not improved after medications, physical therapy, or injections. Additionally, the comparative analysis of outcomes in patients with a history of CRFA or cervical surgery underscores potential advantages of PNS prior to destructive therapies. Future research, ideally in the form of prospective studies with larger cohorts and extended follow-up durations, is warranted to further evaluate long-term outcomes and refine the place of PNS in the treatment algorithm.

## 1. Introduction

Neck pain is a highly prevalent condition, affecting an estimated 203 million individuals in 2020 [1]. Its incidence ranges from 10.4% to 21.3% annually, rendering it the fourth leading cause of disability [2]. Globally, females exhibit a higher age-standardized prevalence than males, with both sexes experiencing peak prevalence between the ages of 45 and 74. Projections indicate that by 2050, the global burden of neck pain will escalate to 269 million cases—a 32.5% increase [1]. This condition imposes substantial direct and indirect economic costs, with healthcare expenditures estimated to exceed USD 686 million, including USD 160 million in direct and USD 527 million in indirect costs [3]. The economic and social impacts of neck pain are expected to intensify, particularly in low- and middle-income countries, underscoring its growing global burden. The etiology of neck pain is multifactorial, with potential origins in the cervical musculature, ligaments, facet joints, intervertebral discs, and spinal nerves. Notably, pain originating from the cervical facet joints accounts for up to 60% of cases [4].

The predominant cause of facet joint pathology is spinal degeneration, or spondylosis, which results from natural wear, abnormal biomechanics, traumatic injuries, repetitive strain, and synovial inflammation [5]. Diagnosis is primarily clinical, with facet-mediated pain typically presenting as axial, non-radicular discomfort that is exacerbated by extension or movements that overload the facet joints [6]. Initial management involves a multimodal conservative approach—including pharmacotherapy, physical therapy, and manual interventions—while interventional procedures, such as diagnostic cervical medial branch nerve (CMBN) blocks, are employed to confirm facet involvement. A positive response to dual diagnostic blocks may then support further treatments, such as cervical radiofrequency ablation (CRFA) [5,7]. Despite the documented efficacy of CRFA, a subset of patients either exhibits a suboptimal response or opts for alternative therapies [8].

Recent interventional advancements have concentrated on the electrical modulation of the CMBN to achieve palliative analgesia. This approach primarily targets the afferent pain signal and interrupts the central sensitization process [9]. One such innovation is a 60-day percutaneously implanted peripheral nerve stimulation (PNS) device with externalized leads (SPRINT, SPR Therapeutics Inc., Cleveland, OH, USA), which is indicated for refractory chronic pain. This system utilizes a temporary fine wire, implantable unilaterally or bilaterally under fluoroscopic or ultrasound guidance. Investigations into its electrical parameters have established a stimulation frequency of 12 Hz and an amplitude between 5 and 20 mA. The device administers cyclical stimulation for six hours daily over the 60-day period, after which the system is removed by the physician, in compliance with Food and Drug Administration (FDA) clearance [10,11]. PNS has demonstrated safety and efficacy across various chronic pain conditions, including spinal and peripheral neuropathies, post-amputation pain, post-surgical pain, and more recently, facial and head pain [12,13,14].

Despite these technological advancements, the literature to date is limited to case reports and abstracts addressing the use of CMBN-targeted PNS for chronic axial neck pain. Consequently, this literature review and case series aim to document the application of this emerging therapeutic modality for patients with chronic axial neck pain refractory to conventional conservative measures.

## 2. Methods

This study comprises a narrative review of the literature on PNS of the cervical spine and a single-center, retrospective case series. A comprehensive narrative review was conducted to contextualize the clinical utility of PNS in managing chronic axial neck pain. A systematic search strategy was developed targeting electronic databases, including PubMed, Embase, and the Cochrane Library. Search terms encompassed “peripheral nerve stimulation”, “cervical medial branch nerves”, and “chronic neck pain”. Studies published in English from database inception until the date of the last search were eligible for inclusion. Articles were initially screened by title and abstract, with full-text reviews performed to assess relevance and quality. Data from selected studies were subsequently extracted and synthesized qualitatively. This review aimed to highlight gaps in the literature and inform the rationale for the subsequent case series.

The case series was of a single-center retrospective nature and evaluated adult subjects (>18 years) with chronic axial neck pain attributable to cervical spondylosis facet arthropathy, absent myelopathy, or radiculopathy. This retrospective study underwent initial review by the institutional Review Board (IRB #00160891) and involved retrospective analysis without identified subject information. Subjects’ data were collected from July 2023 to July 2024. All patients had experienced refractory pain for a minimum duration of six months despite conservative treatments—including medications, physical therapy, chiropractic interventions, injections, and CRFA. Diagnosis was established through clinical evaluation by a pain physician, diagnostic imaging, and all subjects underwent confirmatory cervical medial branch blocks (CMBBs). Patients with and without prior cervical spine surgery or a history of CRFA were included, while exclusion criteria encompassed radicular arm pain and contraindications to PNS placement (e.g., presence of a pacemaker, presence of a spinal cord stimulator, coagulopathy, infection, etc.). Under fluoroscopic guidance, experienced pain physicians performed bilateral percutaneous lead placements targeting the CMBN using the SPR device (Sprint, Cleveland, OH, USA). The selection of lead placement sites was informed by a combination of location of symptoms (higher, middle, or lower cervical spine), physical examination findings, imaging results, and the patient’s response to confirmatory diagnostic medial branch blocks. Subjects with headache and upper cervical spine pain had the occipital nerves and C3 MBN targeted. Subjects with mid–lower cervical spine pain had the cervical MBN targeted based on symptoms related to a facetogenic referred pain pattern to the parascapular region, in combination with a physical exam and imaging finings. Device parameters—including mode, frequency, amplitude, and pulse width—were titrated by clinical specialists to ensure a comfortable sensation and adequate coverage of the painful region. The temporary leads remained in situ for 60 days, after which they were removed upon completion of therapy. Pain and disability outcomes were assessed at baseline and at the 60-day follow-up using the numeric rating scale (NRS) and the Oswestry Disability Index (ODI), respectively. Although interval data were collected when available, standardization across subjects was not uniform. Descriptive statistical methods were employed to summarize the demographic and clinical characteristics of the subject cohort, including means, standard deviations (SDs), frequencies, and percentages, as appropriate. NRS and ODI were specifically evaluated from baseline to last follow-up to establish the minimal clinically important difference (MCID) based on established thresholds, providing clinically relevant context for interpreting patient improvement beyond statistical values [15,16]. Additionally, exploratory analysis was conducted using paired *t*-tests to compare pre- and post-intervention scores, with a two-tailed *p*-value. A *p*-value of <0.05 was considered indicative of statistical significance. Given the novel application of this therapy for this particular indication, we further explored the impact of prior cervical spine surgery and cervical radiofrequency ablation within our cohort.

## 3. Results

### 3.1. Literature Review

Our narrative review found only four studies examining the use of neuromodulation of the CMBN in the treatment of chronic axial neck pain [17,18,19,20]. Of these, two described peripheral nerve field stimulation (PNFS) [17,19], one described subcutaneous PNFS [18], and only one described a single case of temporary 60-day CMBN-targeted PNS [20]. Although both PNS and PFNS employ electrical stimulation for pain neuromodulation, they differ in their mechanisms, indications, procedural techniques, and stimulation settings. PFNS delivers stimulation to a broader array of nerve fibers within the painful region without isolating a single nerve, while 60-day temporary PNS specifically targets an individual peripheral nerve with sensory and motor stimulation with selective large-diameter afferent fiber activation that may reverse changes, leading to a prolonged reduction in pain with peripherally induced reconditioning of the central nervous system [21,22] (Table 1).

Although data regarding the use of PNS in the cervical spine are limited, numerous studies have investigated its application for craniofacial neural targets. This is particularly evident in research focusing on conditions such as occipital neuralgia, cervicogenic headaches, and trigeminal neuralgia. A robust body of clinical evidence—including several randomized controlled trials—has demonstrated that PNS can significantly reduce pain intensity and enhance quality of life in patients suffering from refractory craniofacial pain [25]. Notably, the efficacy of occipital nerve stimulation in chronic migraine treatment has been well substantiated through these trials. Although some studies suggest that PNS may offer more sustained relief compared to pharmacological management or physical therapy, the variability in procedural techniques, targeted neural structures, and the complexity of craniofacial pain syndromes continues to challenge the reproducibility of these outcomes [26,27,28,29,30,31,32,33,34].

### 3.2. Case Series

The case series comprised 9 adult patients with a mean age of 72.4 years, each reporting chronic axial neck pain persisting for over 12 months. Among the participants, five were non-smokers, two were former smokers, and two were current smokers. Only one patient had diabetes, whereas the remaining eight had no such history. The cohort was composed of four males and five females. All patients received a primary diagnosis of cervical spondylosis facet arthropathy, with no evidence of myelopathy or radiculopathy. Regarding PNS placement, the bilateral C6 location was most frequently employed (5 of 10 placements), followed by bilateral C5 (2 of 10 placements), with single placements at bilateral C4, right C3, and the right occipital nerve. No serious adverse events were reported during the study; however, one subject experienced transient skin irritation from the dressing, which resolved following subsequent dressing changes.

Five subjects had a history of CRFA, with three undergoing the procedure within the six months preceding the current intervention. The mean interval between the last CRFA and the temporary PNS was 19.2 months. Notably, 44% of the subjects had previously undergone cervical spine surgery. Table 2 summarizes subjects’ demographics and characteristics. Baseline assessments revealed a mean NRS score of 5.3 and a mean ODI of 40. At the 60-day follow-up, these values improved to a mean NRS of 2.8 and a mean ODI of 20.2, corresponding to a 47.2% reduction in NRS and a 49.5% decrease in ODI from baseline. On average, subjects experienced a 44.4% reduction in pain following the full treatment duration. Both a minimal clinically important difference (MCID) and statistically significant improvements (*p* < 0.05) in pain and disability were observed from baseline to the 60-day follow-up. For the purposes of this study, MCID was defined as a reduction exceeding 1.7 points in the NRS and a reduction exceeding 10 points in the ODI from baseline [15,16]. Specifically, 66% of subjects achieved the MCID for pain reduction, and 77% achieved the MCID for disability reduction. Table 2 details the summary of statistical analysis of outcomes and MCID.

Given the novel application of this therapy for this particular indication, we further explored the impact of prior cervical spine surgery within our cohort. This is the first study to assess the efficacy of PNS in patients who had previously undergone spinal surgery. In prior studies examining the use of PNS for the lumbar medial branch nerve, patients with a history of lumbar spine surgery were excluded [35,36,37,38]. In our cohort, 44% of patients had undergone previous neck surgery. We also examined the influence of prior CRFA in this case series, and 55% of patients had previously undergone CRFA before initiating PNS therapy. Table 3 summarizes these findings.

**Figure 1:**
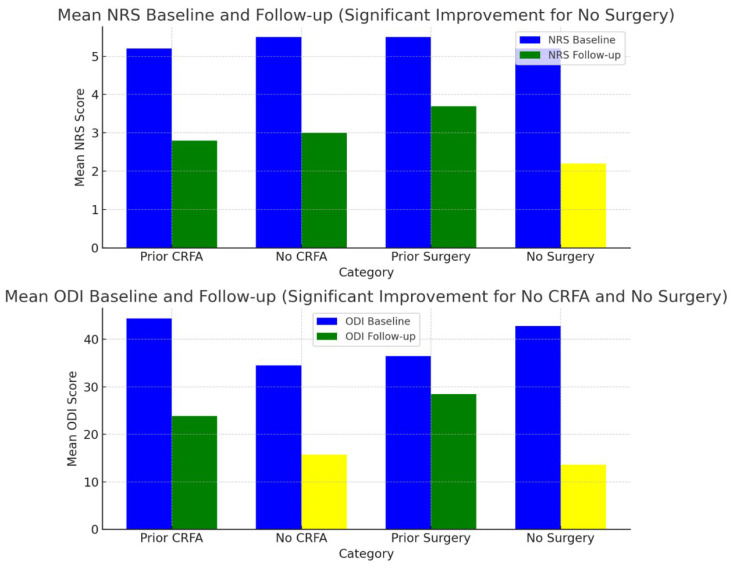
Changes in NRS and ODI comparing prior surgery and CRFA subjects with naive subjects. Yellow bar denotes the only findings that were statistically significant (*p* < 0.05).

Of those with a surgical history, 31.8% experienced a reduction in NRS scores, 21.9% had a decrease in ODI, and a 25% overall improvement in outcomes was seen. In contrast, patients without prior surgery exhibited a more robust response, with a 57% reduction in NRS scores, a 68.2% reduction in ODI, and an average overall improvement of 60%. The less pronounced improvements observed in post-surgery patients may be due to factors such as post-surgical scarring or fibrosis, which could impact the integrity or anatomical location of the CMBN and the placement of surgical hardware. We also examined the influence of prior CRFA in this case series, and 55% of patients had previously undergone CRFA before initiating PNS therapy. Among these patients, we observed a 46.2% reduction in NRS scores, a 46.4% decrease in ODI, and an average overall improvement of 48%. By comparison, patients without a history of CRFA experienced a 45.5% reduction in NRS scores, a 54.3% reduction in ODI, and a 40% overall improvement. When subjects were analyzed based on their history of cervical spine surgery and their naive counterparts, and history of CRFA and their naive counterparts, the only statically significant findings were improvements in disability (ODI) in the surgery-naive and CRFA-naive subjects and improvement in pain (NRS) in the surgery-naive group (Table 4). Figure 1 illustrates these findings. Unlike CRFA, which aims to relieve pain through denervation of the CMBN that innervates arthritic cervical facet joints, PNS is believed to alleviate chronic axial neck pain via a nondestructive neuromodulatory mechanism [39]. While the precise reasons for the differential outcomes are not entirely understood, it is possible that the preservation of the CMBN in patients who did not undergo RFA allowed for more effective neuromodulation, resulting in superior outcomes with PNS therapy.

### 3.3. Procedure Description

**Figure 2:**
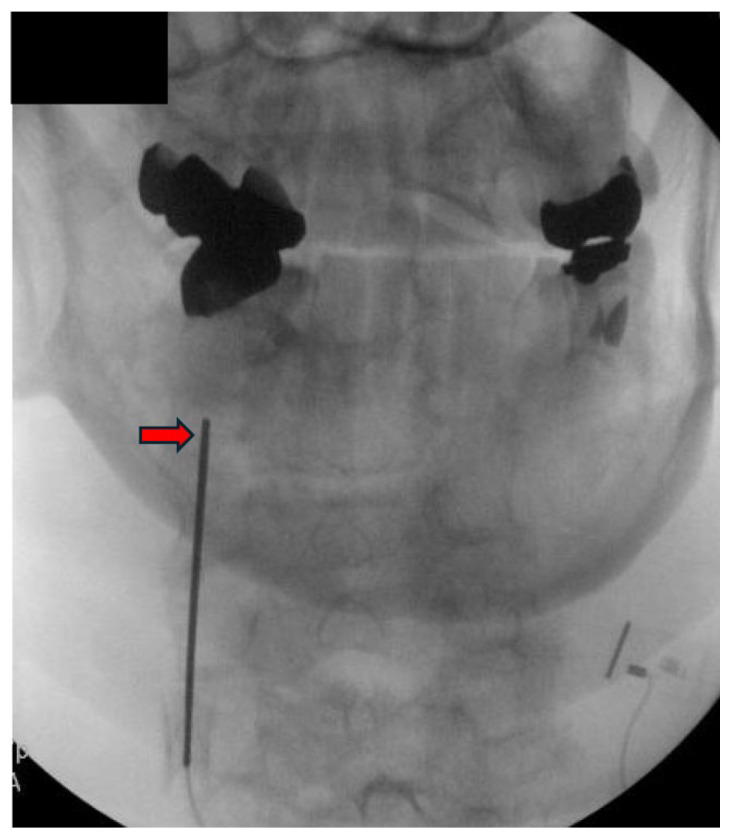
Introducer needle positioned over the mid-cervical articular pillar at C3 under AP fluoroscopic view (red arrow). Image courtesy of Vinicius Tieppo Francio MD.

**Figure 3:**
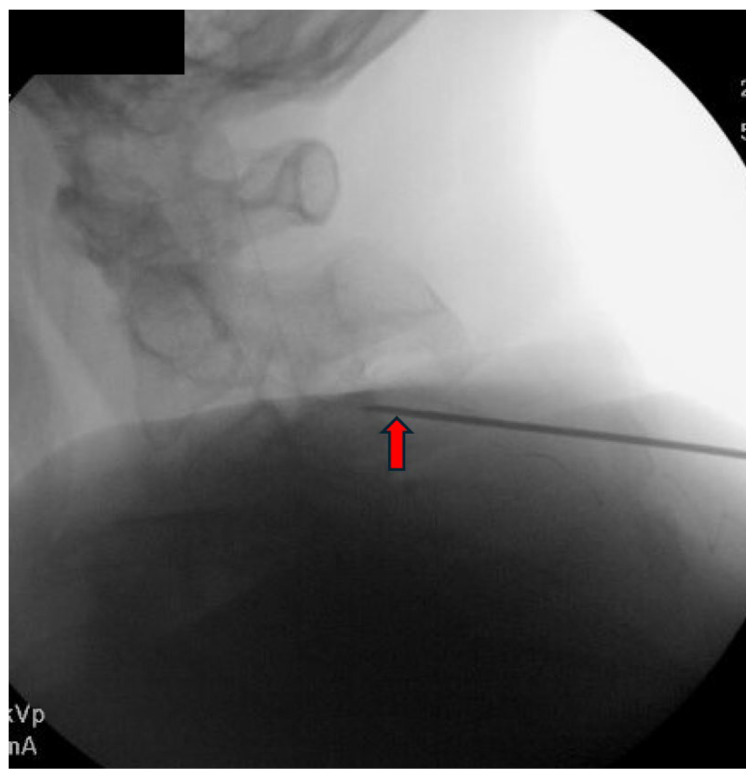
Lateral view confirming introducer needle position over the mid-cervical articular pillar at C3 (red arrow). Image courtesy of Vinicius Tieppo Francio MD.

Appropriate candidates for CMBN PNS are typically patients presenting with chronic neck pain, which may include cases of post-surgical pain that have failed to respond to conservative measures, including CRFA. Absolute contraindications include active infection, untreated coagulopathy, and untreated psychological disorder. The patient is positioned prone with the head maintained in neutral alignment on a soft headrest to prevent cervical rotation or extension. A procedural timeout is performed to confirm the patient’s identity, and the targeted spinal level and laterality. The posterior cervical region is prepped in a sterile fashion. The EPG is secured outside the sterile field. Local anesthesia is administered approximately one to two vertebral levels below the intended target to maintain an inferior to superior trajectory along the cervical articular pillars. The anesthetic should be administered carefully near the final target site to preserve the fidelity for stimulation testing. Under anteroposterior (AP) and lateral fluoroscopic guidance, a 17-gauge sheath is introduced and advanced toward the dorsal aspect of the lateral mass at the desired level. Proper needle position is confirmed in both views, ensuring alignment over the articular pillar (Figure 2 and Figure 3).

**Figure 4:**
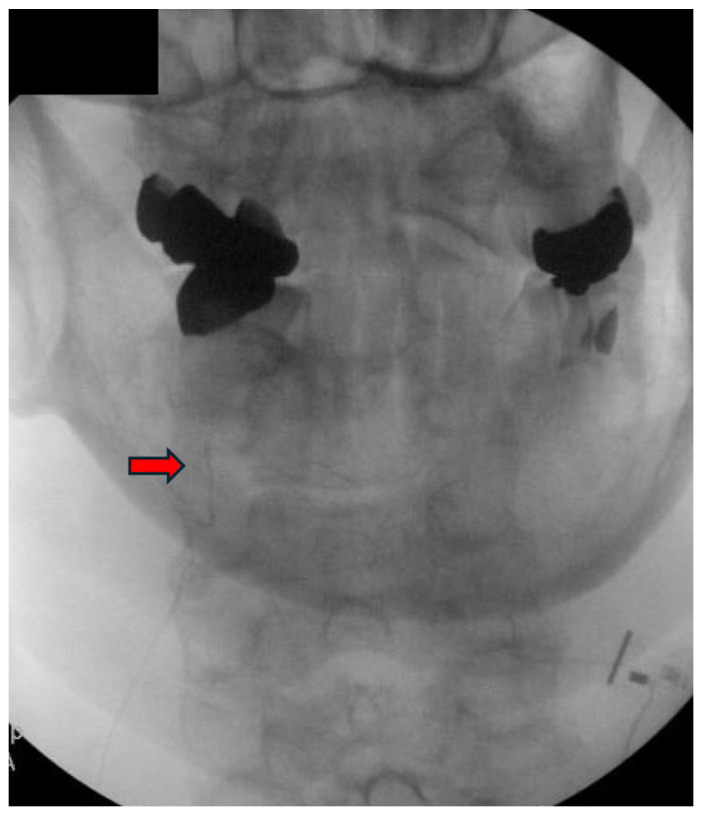
Final lead position confirmed under AP fluoroscopy with distal electrode at the C3 articular pillar (red arrow). Image courtesy of Vinicius Tieppo Francio MD.

**Figure 5:**
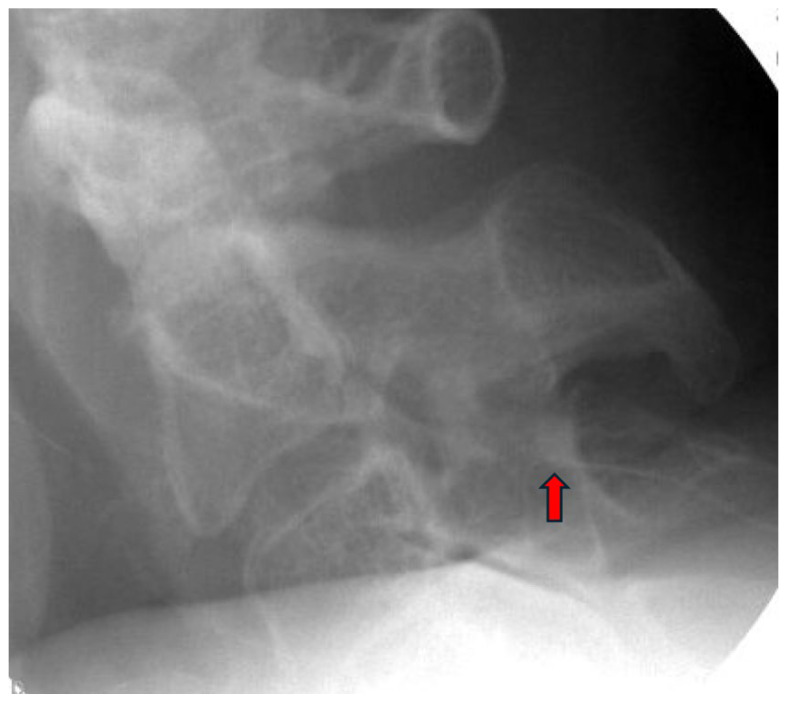
Lateral view of final lead deployment at the C3 articular pillar (red arrow).

Following appropriate sheath placement, a 19-gauge stimulating probe is inserted and connected to the EPG via the test cable. Stimulation is initiated and gradually titrated until the patient reports comfortable paresthesia overlapping the painful region. The position is adjusted as needed to optimize coverage. Once optimal stimulation is achieved, the probe is withdrawn, and the 20-gauge micro-lead introducer is inserted through the sheath. The stimulation lead is advanced to the predetermined depth, and its exposed end is connected to the connector box to reconfirm sensory coverage under low-intensity stimulation. After confirming lead positioning, stimulation is deactivated. The sheath and introducer are withdrawn as a unit while maintaining pressure at the entry site to prevent displacement. The lead is coiled to create strain relief and secured within the connector cradle. Excess lead is trimmed and routed externally to the EPG. Final lead placement is verified fluoroscopically in both AP and lateral views, ensuring the distal electrode lies along the mid-articular pillar (Figure 4 and Figure 5). After implantation, an occlusive dressing is applied, and patients are observed briefly and discharged with clear postoperative instructions. Activity restrictions for 48 to 72 h include avoiding excessive cervical rotation or flexion. The early follow-up appointment is used to assess the effectiveness of stimulation, confirm lead positioning, and manage any emerging complications, such as lead fracture, lead migration, and site infection.

## 4. Discussion

This is among the first studies published in the literature evaluating pain and functional outcomes in patients with chronic axial neck pain following 60 days of temporary PNS targeting the CMBN. The findings demonstrate clinically significant improvements in both pain and disability, with a 47.2% reduction in pain and a 49.2% decrease in disability from baseline observed at the 60-day follow-up. Moreover, 66% of the subjects achieved the minimal clinically important difference (MCID) in pain reduction, while 77% met the MCID for disability reduction.

PNS has undergone remarkable advancements, driven by the introduction of novel neural targets, refined procedural techniques, and the development of miniaturized devices. These innovations have enhanced the precision of nerve targeting and enabled more tailored, condition-specific therapies [40]. Consequently, the clinical adoption of PNS has increased, supported by a growing body of robust evidence, including Level I data, affirming its efficacy across various pain conditions [12]. While this case series presents a novel application of PNS for CMBN, the technique has been extensively employed in treating chronic pain by targeting other neural structures, such as the occipital, genicular, suprascapular, axillary, cluneal, pudendal, transversus abdominis plane, and lumbar medial branch nerves. PNS has shown effectiveness in managing a range of pain conditions, including post-amputation pain, postoperative pain, and intractable neuropathic pain [10,11,12,13,14,35,41,42,43]. Despite the novelty of our findings regarding 60-day PNS therapy for chronic axial neck pain, our results are consistent with those reported in other PNS studies. Yet further prospective studies and randomized controlled trials are needed to validate these preliminary findings and explore long-term efficacy.

In a recent study, Hatheway et al. presented long-term outcomes from the first RCT assessing the efficacy of PNS for the management of peripheral neuralgia/neuropathy, employing a permanent, externally powered micro-implantable pulse generator. The findings of this RCT revealed responder rates ranging from 86% to 89%, accompanied by statistically significant improvements in pain relief, disability, quality of life, and mood, with these positive benefits being maintained over a 12-month follow-up period [44]. However, this study has certain limitations. Notably, participants in the conventional medical therapy group received treatment for only three months. Additionally, a substantial proportion of the study population—approximately 50%—underwent PNS specifically for low back pain, potentially introducing a treatment-specific bias in the overall findings. Furthermore, similar positive outcomes have been reported with temporary, percutaneous PNS therapies targeting a variety of peripheral nerves across multiple chronic pain etiologies. These conditions include chronic shoulder pain, with stimulation targeting the axillary and suprascapular nerves, lower extremity neuropathic pain following amputation, targeting the femoral and sciatic nerves, and low back pain, addressed via stimulation of the lumbar medial branch nerves. Pritzlaff et al.’s recent review of prospective studies investigating the use of percutaneous PNS for chronic pain treatment, which included several RCTs, highlighted emerging evidence that this treatment modality may offer sustained pain relief without the need for a permanent PNS system [45]. Although long-term pain relief was reported by the authors, only 4 of the 16 studies included follow-up periods extending to at least 12 months. Additional limitations include substantial heterogeneity in study design, such as variations in pain severity thresholds for study inclusion, treatment duration, and enrollment criteria. Sample sizes also varied widely, with the largest study including 74 participants and the smallest enrolling fewer than 10 [45]. These conclusions are further supported by real-world data showing similar outcomes [23]. Notably, in a two-year multicenter longitudinal retrospective cohort study, significant reductions in pain intensity were sustained for up to 24 months, suggesting that temporary PNS implants may offer long-lasting pain relief even after the removal of the temporary leads [46]. This enduring relief is thought to arise from long-term modulation and inhibition of central neuroplasticity [36,39,46].

Our study adds new insights to the existing body of literature by demonstrating significant and clinically meaningful improvements in both pain and function following temporary PNS therapy targeting the CMBN in patients with refractory chronic axial neck pain. This is among the first published studies to explore this application of an emerging technology, including its use in individuals with a history of cervical fusion and CRFA. We hope our findings will encourage further studies with larger sample sizes, randomization, and extended follow-up periods, while also promoting the expansion of PNS to additional neural targets. Ultimately, we aim to increase patient access to innovative therapeutic options for chronic neck pain, a highly prevalent and debilitating condition. However, our study has several limitations. First, the retrospective case series design limits the generalizability of our findings, which may not be applicable to other patient populations. Additionally, the absence of a control group and randomization further weakens the strength of the conclusions. Our data are also constrained by a relatively short follow-up period of 60 days, underscoring the need for future research to assess the long-term efficacy of this approach with 3 or 6 months of follow-up data or longer. Several attempts were made to conduct long-term follow-up with study participants, but these efforts were unsuccessful. Medication use could be a potential cofounder factor in our study. Future studies could focus on specific pain phenotypes and evaluate the effects of prior spinal surgeries and CRFA to refine patient selection criteria, improve safety, and enhance clinical outcomes.

## 5. Conclusions

This case series is among the first studies to assess pain and functional outcomes in individuals with chronic axial neck pain following 60-day PNS therapy targeting the CMBN. The results demonstrated clinically significant improvements in both pain and disability at the 60-day follow-up. However, patients with a history of cervical spine surgery or prior CRFA appeared to experience more limited outcomes compared to those who were surgery- and CRFA-naive. These findings suggest that PNS may serve as a promising adjunctive therapy for carefully selected patients with refractory chronic axial neck pain, particularly of facet joint origin. Nonetheless, further research is required, specifically prospective studies with extended follow-up periods, to better evaluate long-term pain and disability outcomes beyond the initial 60-day therapy period.

## Figures and Tables

**Table 1:** Summary of existing literature of peripheral neuromodulation for neck pain.

Author, Year	Study Design	Type of Stimulation	Duration of Stimulation	Results	Comments
Huntoon et al., 2023 [23]	Retrospective Study	PNS	Temporary 60-day	-Out of a total of 6160 patients who underwent PNS in the study, 76 received PNS targeting the CMBN -Nearly 65% of those patients experienced greater than 50% pain relief and/or clinically significant improvement in the Patient Global Impression of Change (PGIC)	-This study was not limited to the CMBN PNS but encompassed a diverse range of chronic pain etiologies -No subgroup analyses of the CMBN PNS group were conducted -The study did not specify the underlying diagnoses or etiologies of neck pain that were treated
Haider et al., 2023 [20]	Case Report	PNS	Temporary 60-day	-A 50% reduction in pain intensity on the NRS was observed, along with subjective improvement in cervical range of motion	-This is the first case report of PNS of the CMBN for the treatment of axial neck pain
Owada et al., 2021 [19]	Case Report	PFNS	Permanent	-The patient reported a 70% decrease in both occipital and axial neck pain; however, this improvement was not quantified using a standardized outcome measure	-This is the first case report of PNFS being used to treat both axial neck and occipital neuralgia
Mainkar et al., 2021 [24]	Case Series	PNS	Temporary 60-day	-One out of the eleven patients (1/11) underwent targeting of the left C8 nerve root for the treatment of post-mastectomy pain and intercostobrachial neuritis -The patient experienced good paresthesia coverage at implant and two days following; however, they developed a rash shortly after and the device was removed	-This study evaluated PNS of the C8 nerve root and not the CMBN
Burgher et al., 2012 [18]	Case Series	PFNS	Permanent	-Ten patients underwent a trial of subcutaneous PFNS, with ten proceeding to permanent implantation -The primary outcome measure was patient-reported pain relief at the last follow-up, with an average improvement of 45%; however, this was not assessed using a standardized outcome measure	-Electrodes were placed in the deep subcutaneous tissue, positioned at the interface between the hypodermis and the first major fascial plane, corresponding to the identified area of pain -The majority of patients in the study had a diagnosis of failed back surgery syndrome, which may limit the generalizability of the findings
Lipov et al., 2009 [17]	Case Report	PFNS	Permanent	-The patient reported 100% pain relief sustained through the nine-month follow-up period; however, this outcome was not evaluated using a standardized or validated assessment tool	-This is the first case report of PNFS being used for axial neck pain

**Table 2:** Summary of patient characteristics, demographics, and results.

Case #	Age	Gender	Smoker	Diabetic	Prior CRFA	Prior Neck Surgery	Level of Intervention	NRS at Baseline	NRS at 60-Day Follow-Up	% Pain Relief Reported	ODI at Baseline	ODI at 60-Day Follow-Up
1	77	Female	No	No	Yes	No	Bilateral C4	4	1	80%	44	8
2	79	Male	Yes	Yes	No	No	Bilateral C6	4	2	70%	22	12
3	81	Male	No	No	No	No	Bilateral C6	6	5	60%	32	4
4	66	Female	No	No	Yes	Yes	Bilateral C6	4	0	50%	40	32
5	92	Female	No	No	No	No	Right C3 and Occipital	5	0	30%	44	21
6	83	Female	Former	No	No	Yes	Bilateral C5	7	5	0%	40	26
7	68	Male	No	No	Yes	Yes	Bilateral C6	3	2	20%	24	24
8	46	Male	Yes	No	Yes	Yes	Bilateral C6	8	8	30%	42	32
9	60	Female	Former	No	Yes	No	Bilateral C5	7	3	60%	72	23

**Table 3:** Statistical analysis of pain, functional outcomes, and minimal clinical important difference. * represents statistically significant values.

	Baseline (Standard Deviation)	60-Day Follow-Up (Standard Deviation)	Minimal Clinical Important Difference Achieved?	95% Confidence Interval (CI)	*p*-Value
Pain	5.3 (SD 1.7)	2.8 (SD 2.6)	Yes, 60% of subjects	−4.6951 to −0.3049	0.02 *
Disability	40.0 (SD 14.5)	20.2 (SD 10.8)	Yes, 70% of subjects	−33.0729 to −6.527	0.006 *

**Table 4:** Summary of results comparing history of CRFA and neck surgery with naive subjects. * represents statistically significant values.

	Total (N)	Mean NRS Baseline (SD)	Mean NRS Follow-Up (SD)	95% Confidence Interval Related to NRS	*p*-Value Related to NRS	Mean ODI Baseline (SD)	Mean ODI Follow-Up (SD)	95% Confidence Interval Related to ODI	*p*-Value Related to ODI
Prior CRFA	5	5.2 (2.1)	2.8 (3.1)	−6.2614 to 1.4614	0.18	44.4 (17.3)	23.9 (9.8)	−41.0048 to 0.0048	0.05
No CRFA	4	5.5 (1.2)	3 (2.4)	−5.7829 to 0.7829	0.11	34.5 (9.7)	15.7 (9.7)	−35.5832 to −2.0168	0.03 *
Prior Surgery	4	5.5 (2.3)	3.7 (3.5)	−6.9239 to 3.3239	0.4	36.5 (8.3)	28.5 (4.1)	−19.3261 to 3.3261	0.13
No Surgery	5	5.2 (1.3)	2.2 (1.9)	−5.3742 to −0.6258	0.01 *	42.8 (18.7)	13.6 (8.2)	−50.2575 to −8.1425	0.01 *

## Data Availability

The original contributions presented in this study are included in the article. Further inquiries can be directed to the corresponding author.

## Abbreviations

CMBN, cervical medial branch nerve; PNS, peripheral nerve stimulation; CRFA, cervical radiofrequency ablation.
